# Quantitative phosphoproteomic profiling of fiber differentiation and initiation in a fiberless mutant of cotton

**DOI:** 10.1186/1471-2164-15-466

**Published:** 2014-06-12

**Authors:** Qifeng Ma, Man Wu, Wenfeng Pei, Haijing Li, Xingli Li, Jinfa Zhang, Jiwen Yu, Shuxun Yu

**Affiliations:** College of Agronomy, Northwest A&F University, Yangling, 712100 China; State Key Laboratory of Cotton Biology, Institute of Cotton Research of CAAS, Anyang, 455000 China; Department of Plant and Environmental Sciences, New Mexico State University, Las Cruces, NM 88003 USA

**Keywords:** *Gossypium hirsutum*, Fuzzless-lintless mutant, Fiber initiation, Phosphoproteomics

## Abstract

**Background:**

The cotton *(Gossypium* spp*.)* fiber cell is an important unicellular model for studying cell differentiation. There is evidence suggesting that phosphorylation is a critical post-translational modification involved in regulation of a wide range of cell activities. Nevertheless, the sites of phosphorylation in *G. hirsutum* and their regulatory roles in fiber cell initiation are largely unknown. In this study, we employed a mass spectrometry-based phosphoproteomics to conduct a global and site-specific phosphoproteome profiling between ovules of a fuzzless-lintless (*fl*) Upland cotton (*G. hirsutum*) mutant and its isogenic parental wild type (WT) at -3 and 0 days post-anthesis (DPA).

**Results:**

A total of 830 phosphopeptides and 1,592 phosphorylation sites from 619 phosphoproteins were identified by iTRAQ (isobaric tags for relative and absolute quantitation). Of these, 76 phosphoproteins and 1,100 phosphorylation sites were identified for the first time after searching the P^3^DB public database using the BLAST program. Among the detected phosphopeptides, 69 were differentially expressed between the *fl* mutant and its WT in ovules at -3 and 0 DPA. An analysis using the Motif-X program uncovered 19 phosphorylation motifs, 8 of which were unique to cotton. A further metabolic pathway analysis revealed that the differentially phosphorylated proteins were involved in signal transduction, protein modification, carbohydrate metabolic processes, and cell cycle and cell proliferation.

**Conclusions:**

Our phosphoproteomics-based research provides the first global overview of phosphorylation during cotton fiber initiation, and also offers a helpful dataset for elucidation of signaling networks in fiber development of *G. hirsutum*.

**Electronic supplementary material:**

The online version of this article (doi: 10.1186/1471-2164-15-466) contains supplementary material, which is available to authorized users.

## Background

Cotton is an important global economic crop that is widely grown for production of textile fiber materials and cottonseed oil [1]. Cotton fibers are single seed trichomes derived from epidermal cells, and their development occurs in four steps: fiber initiation, elongation, secondary cell-wall biosynthesis, and maturation [2, 3]. About 15–25% of epidermal cells differentiate before or on the day of anthesis, and then develop into lint fibers [4]. In addition to their economic value, cotton fiber cells also serve as a classical biological model system for researching mechanisms of plant cell differentiation and elongation [5]. Great progress has been made in illuminating cotton fiber metabolic pathways and molecular mechanisms [6], but most studies have been focused on gene regulation at the transcriptional and, post-transcriptional levels, and a few at the translational level [7–9].

One well-studied cotton line, the Xuzhou 142 fuzzless-lintless (*fl*) mutant, serves as a classical genetic material to investigate molecular events specific to fiber differentiation and initiation. Xuzhou 142 *fl* was found in wild-type Xuzhou 142, a cultivar with lint and fuzz fibers. Although the fiberless phenotype is reportedly controlled by two recessive genes [10], the genetics of cotton fuzzless-lintless fiber production is not well understood. Several studies have been conducted to examine cotton fiber initiation process. Wu et al. [9] used mRNA from 0 day post-anthesis (DPA) ovules of wild-type cotton and six reduced-fiber or fiberless mutants to probe a cotton cDNA microarray covering about 10,410 ovule cDNA clones, and eventually narrowed down the fuzzless-lintless candidate genes to 13. Using Illumina sequencing of transcriptomes of -2 to 1 DPA cotton ovules, Wang et al. [11] compared Xuzhou 142 WT with its *fl* mutant and identified 130 up-regulated genes and 442 down-regulated genes in the WT. In a proteomics analysis, Liu et al. [12] compared -3 DPA and 0 DPA ovules between Xuzhou 142 and its *fl* mutant using a two-dimensional electrophoresis and a tandem mass spectrometry (MS/MS) technology. They found 46 differentially expressed proteins between WT and *fl* ovules. In addition to these studies, as proteomic technology has developed, the number of proteins detectable in a complex protein sample has increased rapidly, and spectral counting in quantitative proteomics has gained recognition [13, 14]. Recent sequencing of the *G. raimondii* genome has also provided excellent tools and resources to study cotton in a greater depth [15]. The data generated by above studies can assist functional annotation of genes and proteins associated with cotton fiber differentiation and initiation.

Despite the importance of cotton fiber differentiation and initiation, the roles of post-translational modifications, especially the reversible phosphorylation of proteins, remain a mystery. Phosphorylation is one of the most important protein post-translational modifications, and is involved in regulating many biological activities. Phosphorylation of specific intracellular proteins/enzymes by protein kinases and dephosphorylation by phosphatases provide information on both activation and deactivation of critical cellular pathways, including regulatory mechanisms of metabolism, cell division, and cell growth and differentiation [16]. In almost all cases, proteins may be phosphorylated on different residues and their properties altered, leading to activation or down-regulation of their activities, modification of subcellular localization stabilities, and consequently alteration of their functions [17]. Eukaryotic proteins are phosphorylated primarily on serine (Ser), threonine (Thr), and tyrosine (Tyr) residues at a ratio of 1800:200:1 [18]. Numerous research projects on phosphoproteomes have generated a large data collection allowing deeper understanding of phosphorylation events in various species, including yeast [19], mice [20], humans [21], Arabidopsis [22], rice [23], and other organisms [24]. Data resources for plant phosphoproteomics produced by these studies are available from public databases such as PhosPhat for Arabidopsis [25], the Plant Protein Phosphorylation DataBase (P^3^DB) [26, 27], and the Medicago Phosphoprotein Database [28].

The iTRAQ (isobaric tags for relative and absolute quantitation) approach is a sensitive, accurate technique for both qualitative and quantitative peptide analysis, and has been successfully used in various proteomic studies, including those involving phosphoproteomics [29–33]. To better understand the molecular mechanisms involving phosphorylated proteins (phosphoproteins) and signaling networks during cotton fiber differentiation and initiation, we investigated the cotton ovule phosphoproteome using a liquid chromatography-tandem mass spectrometry (LC-MS/MS) enriched by titanium dioxide (TiO_2_) affinity chromatography. We used four-plex iTRAQ to compare phosphopeptide levels in ovules at -3 and 0 DPA between the Xuzhou 142 *fl* mutant and its parental WT. We identified 830 phosphorylated peptides from 619 phosphoproteins, providing both quantitative and qualitative information on cotton phosphorylation between *fl* and WT. The information obtained from this research provides valuable resources and novel insights into mechanisms of phosphorylation modification during cotton fiber initiation.

## Methods

### Plant material and chemicals

Plants of *G. hirsutum* L. ‘Xuzhou 142’ with normal fuzz and lint fibers and its isogenic *fl* mutant line were grown in three replications side by side in a field at the Institute of Cotton Research, Chinese Academy of Agricultural Sciences (CAAS), Anyang (E 114°48′, N 36°06′), China. Cotton materials were grown in a normal agronomic field from April to September. Flower buds at -3 DPA and flowers at 0 DPA were collected individually from the 60 plants in morning (9:00–11:00); the ovules were dissected from five bolls cottected in each of 60 plants, frozen in liquid nitrogen, and stored at -80°C until use. For each genotype, we used three biological pools, each with 60 plants grown at similar stages.

An iTRAQ Reagent-4plex Multiplex kit was obtained from Applied Biosystems (Foster City, CA, USA). Ultra-pure HPLC-grade water was produced with a Barnstead Millipore water purification system (Billerica, MA, USA). Titanium dioxide (TiO_2_) beads were obtained from Shimadzu (Kyoto, Japan). Other chemicals were obtained from Sigma-Aldrich (St. Louis, MO, USA).

### Scanning electron microscopy (SEM)

SEM was conducted using a modification of a previously reported procedure [34]. In brief, ovules from -3 DPA and 0 DPA were dissected from WT and *fl* plants, fixed in a solution of 3% formaldehyde and glutaraldehyde in 0.1 M sodium cacodylate buffer (pH 7.4), and rinsed three times in 0.2 M sodium cacodylate buffer (pH 7.4). The ovules were dehydrated in an 30–100% ethanol series for 30 min at each concentration. Ovules were frozen in an Oxford CT 1500 cryotrans system, gold-coated with an ion coater (Eiko IB 3, Tokyo, Japan), and scanned using a Hitachi S-530 scanning electron microscope (Tokyo, Japan) at an accelerating voltage of 15 kV. Ten separate samples were observed and used to produce a representative image.

### Protein extraction, digestion, and iTRAQ labeling

Proteins were isolated in accordance with a protocol described by Wiśniewski [35] with modifications. Plant tissues (1 g) were finely ground, and the powders were precipitated in a 10% (w/v) trichloroacetic acid/acetone solution containing 65 mM dithiothreitol (DTT) at -20°C for 1 h. After extraction, the solution was centrifuged at 10,000 × *g* for 45 min. The supernatant was discarded, and the precipitate was vacuum-dried and solubilized in 1/10 volumes of SDT buffer (4% SDS, 100 mM DTT, and 150 mM Tris–HCl, pH 8.0). The solution was heated in a boiling water bath for 5 min, followed by ultrasonication (10 rounds of 80-W sonication for 10 s with 15-s intervals). Total protein in the supernatant was quantified based on the Bradford method [36].

Protein (300 μg) from three equally pooled biological replicates was diluted with 200 μl UA buffer (8 M Urea and 150 mM Tris–HCl, pH8.0) and subjected to 30-kDa ultrafiltration. Samples were centrifuged at 14,000 × *g* for 15 min; 200 μl UA buffer was then added, followed by centrifugation for an additional 15 min. After addition of 100 μl iodoacetamide (50 mM in UA) and oscillation for 1 min at 600 rpm, the samples were incubated for 30 min in darkness, and then centrifuged at 14,000 × *g* for 10 min. The filters were washed twice with 100 μl UA buffer, and 100 μl dissolution buffer (50 mM triethylammonium bicarbonate at pH 8.5) was added to the filters followed by centrifugation for 10 min. This step was repeated twice, and 40 μl trypsin buffer (2 μg trypsin in 40 μl dissolution buffer) was then added to each filter. The samples were incubated at 37°C for 18 h, and the peptides were collected by centrifugation for 10 min at 14,000 × *g*. This step was repeated twice, and peptide content was determined by spectral density using UV light at 280 nm. About 90 μg of peptides were labeled five times with iTRAQ reagents according to the manufacturer’s protocol (Applied Biosystems). The peptide mixture was vacuum freeze-dried prior to enrichment with TiO_2_ breads.

An overview of the applied analytical strategy using the iTRAQ-based quantitative phosphoproteomic method is presented in Additional file 1: Figure S1. Protein extracts from -3 and 0 DPA developmental stages of WT were respectively labeled with iTRAQ tags 114 and 115, while those of the *fl* mutant were labeled with tags 116 and 117.

### Phosphopeptide enrichment using TiO_2_ breads

The four-plex iTRAQ-labeled peptides were subjected to phosphopeptide enrichment using TiO_2_ beads as described by Larsen et al. [37]. The trypsin-digested peptide mixture was diluted with 1× DHB buffer (0.6% 2, 5-dihydroxybenzoic acid, 16% acetonitrile [ACN], and 0.02% trifluoroacetic acid [TFA]) and added to the TiO_2_ breads. After 40 min of shaking, the TiO_2_ beads were packed into a GELoader tip (Eppendorf, Hamburg, Germany). The column was washed three times with 50 μl washing buffer I (30% ACN and 3% TFA) and then three times with 50 μl washing buffer II (80% ACN and 0.3% TFA). The bound peptides were eluted with 50 μl NH_4_OH, pH 10.5, and then vacuum freeze-dried. The lyophilized phosphopeptides were dissolved in 0.1% formic acid prior to MS analysis.

### Mass spectrometry

Phosphopeptides were subjected to capillary LC-MS/MS using an automated Easy-nLC 1000 system coupled to a Q-Exactive mass spectrometer (Thermo Fisher Scientific, San Jose, CA, USA). A pre-column (20 mm × 100 μm; 5 μm-C18) and an analytical column (250 mm × 75 μm; 3 μm-C18) were used (Thermo Fisher Scientific) with mobile phases A (0.1% formic acid in water) and B (0.1% formic acid in 84% ACN). The phosphopeptides were separated at a flow rate of 250 nl min^-1^ using the following gradient: 0–55% mobile phase B from 0–220 min, 55–100% mobile phase B from 220–228 min, and 100% mobile phase B from 228–240 min. Data-dependent mass spectra were acquired for 240 min. The full MS surveys were collected over a mass-to-charge ratio (m/z) range of 300–1,800, with the resolution set to 70,000 at m/z 200. For MS/MS, we used a resolution of 17,500 at m/z 200, with an isolation window of 2 m/z.

### Database search and quantification

Mascot 2.2 (Matrix Science, Boston, MA, USA) and Proteome Discoverer 1.3 (Thermo Fisher Scientific) software were used to simultaneously identify and quantify phosphoproteins [38–41] based on two combined databases derived from *G. raimondii* (40,976 entries) [15] and the CGI (cotton gene index) database from DFCI (CGI.release-11.zip; 117,992 entries) [42].

The iTRAQ quantification workflow was performed essentially as reported previously [41] (Additional file 1: Figure S1). After estimating the protein concentration of each sample, proteins were digested using a trypsin enzyme to produce proteolytic peptides. Each peptide was labeled with a different iTRAQ reagent and then mixed. The combined peptide mixture was analyzed by LC-MS/MS for both identification and quantification. The sequence of a peptide was determined from the product ions that were generated from cleavage about peptide interresidue bonds using Mascot 2.2. The relative quantity of a peptide among the different samples was determined by comparing the intensities of reporter ion signals also present in the MS/MS scan using the Proteome Discoverer 1.3 software.

The raw files were searched individually with Mascot 2.2 using the following search parameters: selection only of tryptic peptides with two missed cleavages, peptide mass tolerance of ± 20 ppm, and fragment mass tolerance = 0.1 Da. Proteome Discoverer 1.3 software was used to extract the peak intensity of each expected iTRAQ reporter ion from each analyzed fragmentation spectrum. The search parameters were as follows: peptide false discovery rate ≤ 0.01, use only unique peptides, reject all quantification values if not all quantification channels are present, normalize on protein median, normalize all peptide ratios by the median protein ratio, and median protein ratio = 1 after normalization [41]. For each phosphorylation site on the phosphopeptides, Phosphorylation site probabilities were set above 75%, indicating that the site is truly phosphorylated, and Phosphorylation site score was set above 50, indicating a good peptide spectral match [43].

### Bioinformatics and motif analyses

To investigate amino acid frequencies around each identified pSer, pThr, and pTyr site, the 12 surrounding amino acids were retrieved to generate a list of “phosphor-13-mers” using a BioPerl script [44]. In the case of C- and N-terminal peptides, the sequences were completed to phosphor-13-mers with the required number of “X”s, where X indicates any amino acid. Phosphor-13-mers amino acid frequencies surrounding the three amino acid phosphorylation sites (phosphosites) were completed using the WebLogo server [45, 46], and their motifs were extracted using the Motif-X algorithm [47]. Phosphopeptide sequences for these phosphosites were pre-aligned using a custom Perl script, and the pre-aligned phosphor-13-mers from cotton (1,592 phosphosites) and nine other species (47,923 phosphosites) were submitted to the Motif-X algorithm as a foreground process. Because of upload restrictions, the database generated by combining 15,070 random protein sequences derived from the nine species (from the P^3^DB database) and cotton (10 Mb) generated with a custom Perl script was submitted as background. Motif-X default settings of width = 13, occurrence = 20, and significance = 0.000001 were used for pSer and pThr. For pTyr, less stringent settings of width = 13, occurrence = 2, and significance = 0.0005 were used because only 18 peptides contained localized pTyr motifs. Motif identification was carried out using CompariMotif [48] and the PhosphoMotif Finder database [49]. Phosphoproteins and phosphor-13-mer sequences were compared against the P^3^DB database (9 species, 16,477 phosphoproteins, and 47,923 phosphosites) to assess their novelty.

The Blast2GO suite [50] was applied to annotate identified protein sequences. Phosphoprotein functional classification was conducted using Gene Ontology (GO), GO-Enzyme-Code, and other search tools. The Batch sequence search tool of Pfam 27.0 (14,831 families) was used to obtain phosphoprotein domains [51]. The Plant Transcription Factor database (83 species, 129,288 transcription factors) was downloaded from the Center for Bioinformatics [52] and used to identify transcription factors [53]. To search for homologs, the identified phosphoproteins were analyzed by local blast tool in the software package ncbi-blast-2.2.26 + -win32.exe [54] against a reference database downloaded from P^3^DB databases. For multiple sequence alignment, ClustalX2 was used with default parameter settings [55]. The aligned sequences were further analyzed using the MEGA5 program [56]. For pathway enrichment analysis, the differentially phosphorylated proteins were mapped to the terms in the KEGG (Kyoto Encyclopedia of Genes and Genomes) database by using the KOBAS 2.0 (KEGG Orthology-Based Annotation System) program [57]. KEGG pathways with corrected p values ≤ 0.05 were considered to be statistically enriched in cotton fiber initiation.

## Results

### Phosphopeptide identification

When fiber development of the WT and its *fl* mutant was examined by SEM (Additional file 1: Figure S1), no obvious differences were observed between the WT and *fl* at -3 DPA. The ovule surfaces were flat, and the epidermal cells were dotted with stomata. On the day of anthesis, numerous cells destined to become fibers had already started to balloon out from the epidermis on the WT; in contrast, the *fl* mutant had no fiber initiation, as this phenomenon was not observed.

Based on the criteria given in Methods, 830 phosphopeptides and 1,592 individual phosphosites originating from 619 proteins were identified from ovules of *fl* and its parental WT at -3 and 0 DPA (Table 1). The spectra representing all phosphopeptides and the original data were shown in Additional file 2. Of the 1,592 non-redundant phosphosites, 89.4% were phosphorylated at serine, 9.5% at threonine, and 1.1% at tyrosine residues. This finding is consistent with previous reports in other plants: 88% pSer, 11% pThr, and 1% pTyr in Arabidopsis, and 89.3% pSer, 10.2% pThr, and 0.5% pTyr in soybean [30, 58]. A detailed examination of the phosphoproteomic data revealed that 33.4%, 48.3%, and 18.3% of the 830 unique phosphopeptides were singly, doubly, and multiply phosphorylated, respectively (Additional file 3: Table S1, sheet 1). These values are very different from those reported for Arabidopsis (80.9%, 19.1%, and 0%) and soybean (95%, 5%, and 0%) [22, 30], but are more or less consistent with *Saccharomyces cerevisiae* (27.8%, 67.1%, and 5.1%) [19]. This difference might be explained by the use of different methodologies or biological systems, where each tissue and organism under a particular environment may have a special phosphoproteome profile.

**Table 1:** **Numbers of phosphoproteins, phosphopeptides, and phosphorylation sites identified in this study**

Listing	Number
Phosphoproteins	619
Phosphopeptides	830
Phosphopeptides (single:doubleness:mulriple)	277:401:152
Phosphorylation sites	1592
Phosphorylation sites (Ser:Thr:Tyr)	1423:151:18

**Figure 1:**
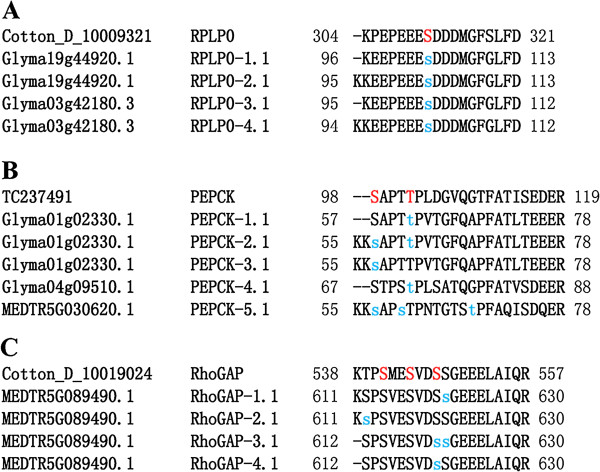
**Conservation of phosphosites between cotton proteins and homologs in nine other plant species.** Phosphosites identified in this study are indicated in red; previously identified phosphosites are represented by lower-case blue letters. **A**: Phosphosites conserved across the plant 60s acidic ribosomal protein P0 (RPLP0). **B**: Phosphosites conserved across the plant phosphoenolpyruvate carboxykinase (PEPCK). **C**: Phosphosites conserved across the plant Rho GTPase activation protein (RhoGAP).

We compared phosphorylation patterns of orthologous phosphosites between cotton and nine other species (from P^3^DB) to analyze phosphosite conservation. Phosphosites in cotton that were absent from their equivalent phosphoproteins in other plant species were considered to be novel. Among the identified phosphosites, 875 were conserved across the nine species, but only 492 were phosphorylated in their P^3^DB orthologs (Table 2 and Additional file 4: Table S2). For example, 60s acidic ribosomal protein P0 (RPLP0, D-10009321) considered as an important protein during the fiber initiation was well conserved [12], and its homologs were phosphorylated (Figure 1A). In contrast, another important protein in fiber initiation named phosphoenolpyruvate carboxykinase (PEPCK, TC237491) had two phosphosites [9], each having homologs (Figure 1B). We also identified two previously unknown phosphosites at the Rho GTPase activation protein (RhoGAP, D-10019024) N-terminus: Ser541 and Ser544 [59], which were well conserved across *Medicago truncatula* but only present in this plant as non-phosphorylated residues. In addition, only cotton phosphopeptide contained all three phosphosites simultaneously (Figure 1C). In total, nearly one-third of orthologs (492; 30.9%) were phosphorylated at equivalent sites that have been previously reported; the majority (1,100; 69.1%) of identified phosphosites, however, were novel. These results also indicate that phosphosites are conserved in a similar manner across different plant species.

**Table 2:** **Comparison of conserved phosphosites in cotton and species in the P** ^**3**^ **DB database**

	Number of phosphorylation sites		
	Conservation of phosphosites (%)	Phosphosites in 9 species counterparts	
		Described (%)	Undescribed (%)
pS	759 (86.7)	436 (88.6)	323 (84.3)
pT	105 (12.0)	50 (10.2)	55 (14.4)
pY	11 (1.3)	6 (1.2)	5 (1.3)
All	875 (100)	492 (56.2)	383 (43.8)

To evaluate whether phosphosites were concentrated in conserved domains, we performed Pfam searches to obtain domain information for 473 of the 619 phosphoproteins (Additional file 5: Table S3, sheet 1). These data suggested that 73.1% of the phosphosites (885 sites) were located outside of conserved domains (Table 3; Additional file 5: Table S3, sheet 2). The finding that most phosphorylation occurs outside of conserved domains is consistent with previous results [60, 61]. Interestingly, nearly one-third (31.9%) of pThrs were found to be located in conserved domains. These data indicate that pThrs may have more impact on domain-associated functions compared with pSers and pTyrs.

**Table 3:** **Location of phosphorylation sites on characterized phosphoprotein domains**

	Number of phosphorylation sites		
	Pfam domain		Total (%)
	ON (%)	OUT (%)	
pS	284 (26.4)	792 (73.6)	1076 (100)
pT	37 (31.9)	79 (68.1)	116 (100)
pY	4 (22.2)	14 (77.8)	18 (100)
All	325 (26.9)	885 (73.1)	1210 (100)

### Phosphoproteome characterization and classification

**Figure 2:**
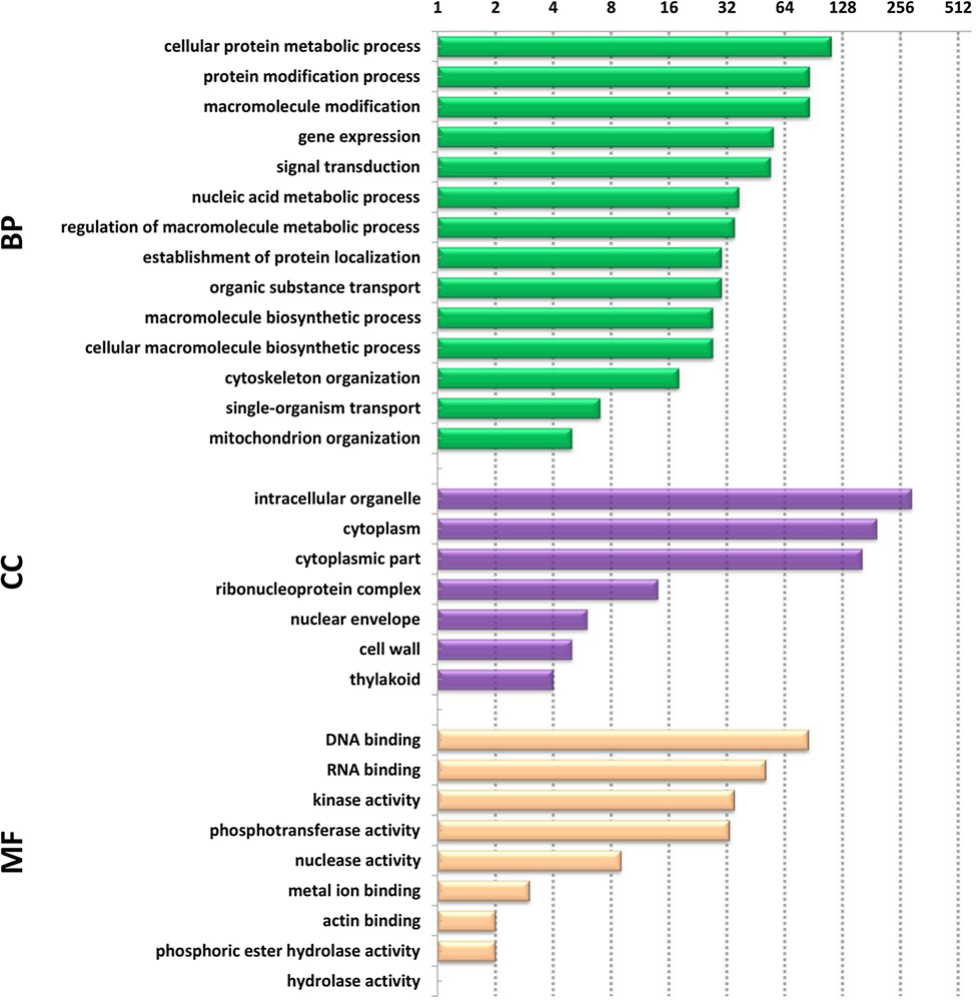
**Distribution of phosphoprotein Gene Ontology functional classifications.** BP: biological process, CC: Cellular component, MF: molecular function.

We used the Blast2GO program to annotate and classify proteins into biological process (BP), cellular component (CC), and molecular function (MF) categories. Sequences were searched against the non-redundant (NR) protein database. Functions and annotations of these predicted phosphoproteins are shown in Additional file 3: Table S1 (sheet 2). The majority of the proteins in our dataset were annotated, with only 29 cases having no annotation information (classified as “unknown”). Among the 619 identified phosphoproteins, information related to BP, CC, and MF was obtained for 351, 370, and 383 phosphoproteins, respectively (Figure 2; Additional file 6: Table S4). In this study, most of the identified phosphoproteins were involved in binding and catalytic activity, consistent with the findings of a previous investigation [58].

To determine if the 619 phosphoproteins identified in our study are commonly found or novel, we searched our dataset against the P^3^DB database, a repository for plant protein phosphorylation site data. P^3^DB currently hosts protein phosphorylation data for nine species from 32 experimental studies, comprising 16,477 phosphoproteins and 47,923 phosphosites. Of our 619 identified phosphoproteins, 543 showed homology to phosphoproteins in P^3^DB, while 76 were novel (Additional file 7: Table S5). These newly identified phosphoproteins may prove useful for identifying components of phosphorylation-dependent signal cascades and for estimating the function of phosphorylation events in response to specific environment signals. The 619 phosphoproteins were also searched against the Plant Transcription Factor Database. As shown in Additional file 8: Table S6, 109 of these phosphoproteins corresponded to transcription factors. This result is consistent with previous studies demonstrating that regulatory proteins, such as transcription factors and kinases, are more often subjected to post-translational regulation via phosphorylation than are metabolic enzymes [60, 62].

### Phosphopeptide motif discovery

**Figure 3:**
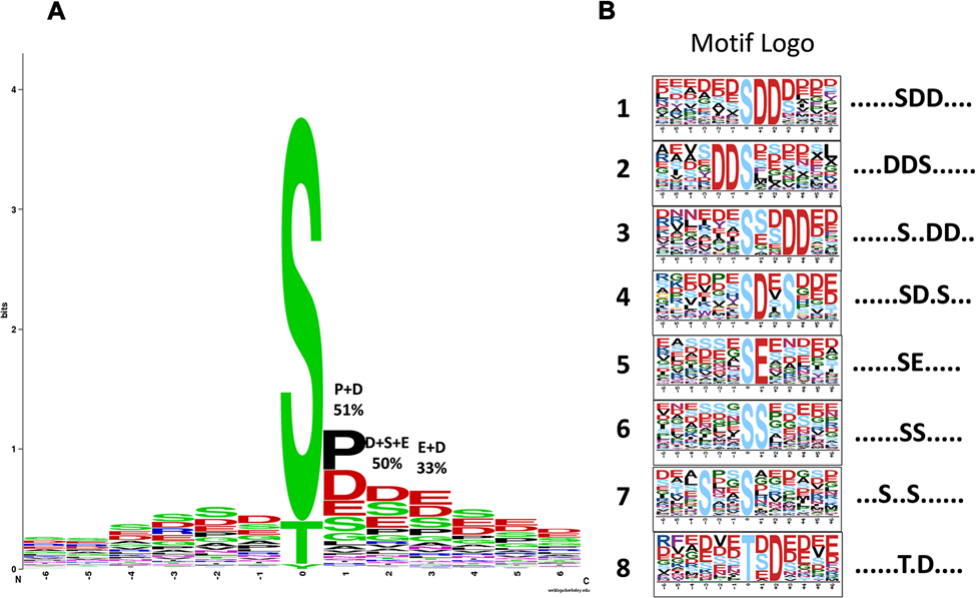
**WebLogo analysis of identified phosphorylation sites and extraction-enriched phosphorylation motifs. A**: Frequency distribution of amino acid residues surrounding phosphorylation sites at positions -6 to +6. **B**: Motifs extracted from the phosphopeptide dataset.

To evaluate sequence conservation at phosphosites, we used WebLogo to generate sequence logos that were graphical representations of patterns within multiple sequence alignments (Figure 3A; Additional file 9: Table S7). The frequency of amino acid residue occurrence at three key positions — n + 1 (proline: P and aspartic: D), n + 2 (aspartic: D, serine: S and Glutamic acid: E), and n + 3 (Glutamic acid: E and aspartic: D) — reached 51%, 50%, and 33%, respectively. We therefore deduced that the sequence pSer/pThr-P/D-D/S/E-E/D is the conserved phosphosite motif. This result may serve as a clue for identifying targets of protein kinases in large-scale phosphopeptide analyses.

Given that protein phosphorylation seems to be the most important proteotype feature, motifs associated with localized phosphosites were identified using Motif-X. To compare potential consensus sequences among different plant species, all phosphopeptides from our study, as well as phosphopeptides from nine other species in P^3^DB, were used for motif extraction against a background database generated by combining 15,070 random protein sequences derived from the genomes of the 10 species (Additional file 10: Table S8, sheet 6). Compared with the 189 phosphorylation motifs detected in the other nine species, 17 pSer motifs and 2 pThr motifs were identified in our cotton dataset. All identified phosphorylation motifs are listed in Additional file 10: Table S8. The 17 pSer motifs could be divided into three major categories: pro-directed, basic, and acidic. Acidic motifs accounted for 11 of the 17 identified pSer motifs, and nearly 65% of Ser phosphopeptides possessed this motif (Table 4). This result was confirmed by a WebLogo alignment of all identified phosphor-13-mer phosphosites (Figure 3A). These results suggest that acidic kinases may be the major kinase group involved in phosphorylation of the identified phosphoproteins during cotton fiber initiation. Among 19 phosphorylation motifs identified in our data, we found 7 distinct motifs in the PhosphoMotif Finder database and 8 motifs unique to cotton (Figure 3B). Four motifs, ......SDD...., ....DDS......, ......S..DD.., and ......SE....., belonged to casein kinase 2. Casein kinase 2 is involved in cell cycle control, DNA repair, circadian rhythm regulation, and other metabolic pathways [63]. The ......SD.S… phosphosite motif resembled a known motif in transforming growth factor-β (TGFB) receptor kinase, which is involved in cell growth, cell differentiation, apoptosis, cellular homeostasis, and other cellular functions [64]. The ......SS..... motif showed high similarity to a motif found in AKT kinase (also known as protein kinase B) family members, which are serine/threonine-specific protein kinases that play key roles in multiple cellular processes such as glucose metabolism, apoptosis, cell proliferation, transcription, and cell migration [65]. The …S..S...... motif was similar to that of extracellular-signal-regulated kinases (ERKs), which are classical mitogen-activated protein kinases (MAPs) involved in various functions, including regulation of meiosis, mitosis, and post-mitotic processes in differentiated cells [66].

**Table 4:** **The description of the identified phosphorylation motifs**

NO	Cotton motif	Novel or not	Motif pattern	Motif class
1	......SP.....	No	sP	Pro-directed
2	......SD.E…	No	sX[D/E]	Acidic
3	......SDD....	Novel	sX[D/E]	Acidic
4	......SD.D…	No	sX[D/E]	Acidic
5	......SE.E…	No	sX[D/E]	Acidic
6	......S.D.E..	No	sXX[D/E]	Acidic
7	....DDS......	Novel	[D/E]s	Acidic
8	......S..DD..	Novel	sXX[D/E]	Acidic
9	......S.....X	No	unknown	unknown
10	.....DS......	No	[D/E]s	Acidic
11	…R..S......	No	RXXs	Basic
12	......SD.S…	Novel	s[D/E]	Acidic
13	......S.D....	No	sX[D/E]	Acidic
14	......SE.....	Novel	s[D/E]	Acidic
15	......S.SP…	No	unknown	unknown
16	......SS.....	Novel	unknown	unknown
17	…S..S......	Novel	unknown	unknown
18	......TP.....	No	unknown	unknown
19	......T.D....	Novel	unknown	unknown

### Analysis of differentially phosphorylated proteins

**Figure 4:**
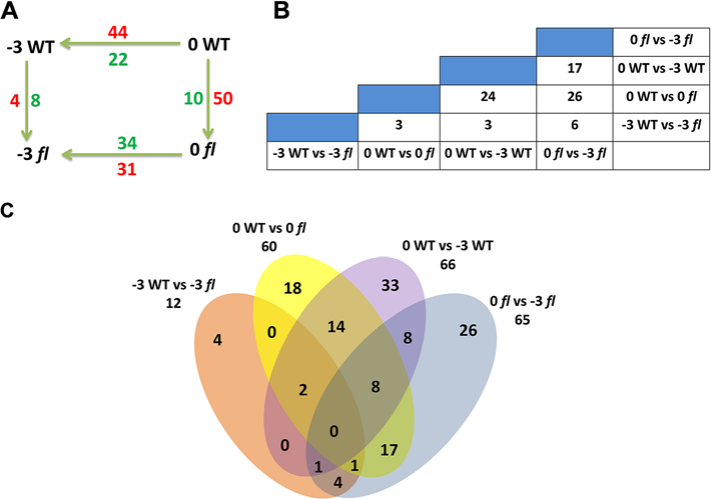
**Summary of differentially phosphorylated proteins identified in this study. A**: Number of phosphoproteins differentially expressed during fiber development within and between WT and *fl* ovules. Numbers above and below arrows denote numbers of phosphoproteins differentially expressed for the specified comparison. For example, between stages -3 and 0 DPA in the WT, 44 phosphoproteins were up-regulated (red) and 22 were down-regulated (blue) at 0 DPA. Similarly, between WT and *fl* at -3 DPA, 4 phosphoproteins were up-regulated and 8 phosphoproteins were down-regulated in the WT. **B**: Numbers at column-row intersections are the number of differentially phosphorylated proteins common to the four tissues. **C**: Venn diagram showing the number of differentially phosphorylated proteins shared between phosphoproteomes of the different four tissues.

Phosphopeptides were considered to be significantly differentially regulated when their levels differed based on a cutoff value of *p* < 0.05 with Student *t-test* and ANOVA analysis. The fold-change cutoff value for up-regulated or down-regulated phosphorylation activity was 1.5-fold. In the four datasets, we found that quantities of phosphoproteins were differentially expressed between -3 DPA WT and -3 DPA *fl,* and between 0 DPA WT and 0 DPA *fl*. Between -3 DPA WT and 0 DPA WT, 66 phosphoproteins were differently expressed. Of these, 44 were up-regulated and 22 were down-regulated in 0 DPA WT compared with -3 DPA WT, suggesting that many phosphoproteins were enriched during the transition from fiber differentiation to initiation. In contrast, 31 were up-regulated and 34 were down-regulated in 0 DPA *fl* compared with -3 DPA *fl*. Similarly, between -3 DPA and 0 DPA in WT and *fl*, 4 and 50 phosphoproteins, respectively, were up-regulated in the WT (Figure 4A; Additional file 11: Table S9). We further classified differential patterns of phosphoprotein expression using the following diagrams (Figure 4B; Figure 4C). For example, the abundances of 69 phosphoproteins were found to change significantly at one or two time points between WT and *fl* (i.e., -3 DPA WT vs. -3 DPA *fl* and 0 DPA WT vs. 0 DPA *fl*). Among these 69 phosphoproteins, the abundances of 3 were significantly changed at both -3 and 0 DPA in both genotypes, whereas the abundance of 9 and 57, respectively, varied significantly only at either -3 DPA or 0 DPA (Figure 4C; Additional file 11: Table S9). Of the 3 phosphoproteins differentially expressed at both time points, 2 were up-regulated at 0 DPA but down-regulated at -3 DPA in the WT; the rest phosphoprotein exhibited the same trend at both time points. The 69 differentially phosphorylated proteins were classified into 6 functional categories based on their predicted molecular functions (Additional file 12: Figure S2 and Additional file 13: Table S10). The largest functional categories were DNA binding, Nucleotide binding, RNA binding, Kinase activity, Phosphotransferase activity and Metal ion binding. The significantly enriched pathways were also analyzed by KOBAS software suite. Four biochemical pathways, such as RNA transport, Herpes simplex infection, Spliceosome and Gap junction, were enriched (Corrected P-Value < 0.05) during the fiber initiation period (Additional file 14: Table S11).

## Discussion

### Differentially phosphorylated proteins involved in signal transduction

We found four differentially phosphorylated proteins (CK640461, TC229918, D-10009824, and D-10019024) related to signal transduction, of which three were up-regulated and one was down-regulated in the WT. CK640461 belongs to the small-GTPase superfamily, comprising Rab, Ran, Arf, and Rho GTPases in Arabidopsis. The different Rab GTPases are localized to the cytosolic face of specific intracellular membranes, where they function as regulators of distinct steps in membrane traffic pathways. The run and tbc1 domain-containing protein (CK640461) has Rab GTPase activity and may regulate vesicle formation, actin- and tubulin-dependent vesicle movement, and membrane fusion [67, 68]. Genetic evidence suggests that GTPases of the Rho class (also called ROPs) are involved in spatial regulation of reactive oxygen species (ROS) production and growth [69]. We also found one Rho GTPase activation protein (D-10019024); the activities of Rho-GTPases are negatively controlled by a group of proteins called Rho-GDP dissociation inhibitors (RhoGDIs) [70]. Loss of function of one member of this family results in both spatially deregulated ROS accumulation and hair outgrowth. However, a constitutively active form of a cotton small GTPase highly expressed during cotton fiber development has been shown to induce ROS production in cultured Arabidopsis and soybean cells [59]. This result also suggests that Rho GTPase can perform its role during the rapid elongation of cotton fibers. IQ-domain 32-like protein (TC229918) may be involved with calmodulin specificity. The IQ-domain is approximately 25 amino acids in length and forms an amphiphilic seven-turn α-helix capable of binding calmodulin in a Ca^2+^-independent manner. Calcium-mediated singnal transduction plays crucial roles in plant growth, especially in tip growth [5]. A highly Ca^2+^ concentration can be observed in the tips of root hairs [71]. Obvious inhibition of fiber growth also occurs when cotton ovules are cultured in the absence of exogenous Ca^2+^ ions [72].Transcription of protein-coding genes by RNA polymerase II (RNAP II) is facilitated by a multitude of transcription factors [73]. Elongation is highlighted as a central process that coordinates multiple stages during mRNA biogenesis and maturation [74]. Transcription elongation factors (D-10009824) are thought to increase RNAP II productivity by facilitating chromatin passage and mRNA processing.

### Differentially phosphorylated proteins associated with protein modification

A protein is made up of amino acid chains, i.e., one or more polypeptides. Post-translational modification, including polypeptide folding, cleavage, and other processes, is one of the steps of protein biosynthesis. A novel phosphoprotein, compared with those of other examined species, was identified in this study: E3 ubiquitin-protein ligase HOS1 phosphoprotein (CO096048). This protein negatively regulates cold signal transduction, and its mutant flowers early [75]. Cyclophilin-like peptidyl-prolyl cis-trans isomerase family protein (D-10005130, PPIase) can accelerate protein folding, and also has protein chaperone-like functions. This protein contains a common cyclophilin-like domain (CLD) and other domains, which are important for selection of protein substrates and subcellular compartmentalization [76]. The formation of a decapping complex comprising Decapping 5 (TC240176, DCP5), which occurs inside processing P-bodies, represents an irreversible step in mRNA degradation. Knockdown mutants of DCP5 share abnormalities in postembryonic development [77]. DCP5 may therefore play an important role in mRNA decapping during postembryonic development. We also identified two protein kinases and two histone deacetylase, all of which were up-regulated in the WT: serine threonine-protein kinase prp4 (D-10033485), g-type lectin s-receptor-like serine threonine-protein kinase sd2-5-like (D-10037115) and histone deacetylase (TC230548, TC232936). A serine/threonine protein kinase is a kinase enzyme that phosphorylates the OH group of serine or threonine. Serine threonine-protein kinase prp4 plays a role in the regulation of pre-mRNA splicing codes and also is essential for yeast growth [78]. Histone deacetylases are a class of enzymes that allow the histones to wrap the DNA more tightly and then regulate DNA expression by acetylation and de-acetylation. In Arabidopsis, histone deacetylase 18 can exhibit altered hair and non-hair epidermal cell patterning [79].

### Differentially phosphorylated proteins involved in carbohydrate metabolism

Two phosphoproteins in this study, both down-regulated in the WT, were identified as especially enriched in the sucrose metabolism pathway. Generally, sucrose synthase (SUS) is a key player in plant sucrose catabolism, and, like invertases (D-10036040), catalyzes the reversible conversion of sucrose and UDP into fructose and UDP-glucose [80]. Sucrose has recently been recognized to have important hormone-like functions as a primary messenger in signal transduction. Additionally, sucrose molecules regulate gene expression at transcriptional and post-transcriptional levels [81, 82]. Increasing evidence indicates that SUS is essential for cell wall thickening and cotton fiber cell development [83–85]. *Sus* is preferentially expressed in elongating fiber cells, and antisense suppression of *Sus* expression reduces hexose levels, leading to a fiberless phenotype [86, 87]. UDP-glucose 6-dehydrogenase (TC263488) is an enzyme encoded by the *UGDH* gene. This enzyme converts UDP-glucose to UDP-glucuronate, which is readily incorporated into the pectin fraction of cell wall preparations and can significantly improve fiber growth during ovule culture [88].

### Differentially phosphorylated proteins related to the cell cycle and cell proliferation

Seven up-regulated phosphoproteins identified in the WT—serrate RNA effector molecule-like (D-10014027), vacuolar import/degradation protein (D-10025850), lysine-specific histone demethylase 1 isoform 3 (D-10036681), uncharacterized protein (D-10037099), ROP interactive partner 5 isoform 4 (DW225237), WD repeat-containing protein 70-like (ES794679) and pre-rRNA processing protein esf1-like (TC233572)—are involved in the regulation of cell cycle patterns and cell proliferation development.

Serrate RNA effector molecule protein belongs to the ARS2 family and contains one C2H2-type zinc finger domain. This protein expresses in shoot meristems and in emerging organ primordia throughout development. Its mutant displays defects in shoot and leaf development or death during embryogenesis in Arabidopsis [89]. Vacuolar import/degradation protein is involved in the negative regulation of gluconeogenesis. This protein is required for both proteosome-dependent and vacuolar catabolite degradation of fructose-1,6-bisphosphatase (FBPase), where it probably regulate FBPase targeting from the FBPase-containing vesicles to the vacuole [90]. Lysine-specific demethylase 1 protein is a flavin-dependent monoamine oxidase, which can demethylate mono- and di-methylated lysines. This protein is a component of several histone deacetylase complexes, though it silences genes by functioning as a histone demethylase. In a certain yeast strain, *Saccharomyces cerevisiae*, to become methylated causes a delay in the mitotic cell cycle [91]. ROP interactive partner 5 isoform 4 is a putative Rho protein effector, interacting specifically with the active form of ROPs (Rho proteins of plants), expresses in the root and shoot apex in Arabidopsis [92]. WD repeat-containing protein 70-like protein can express with the pollen germination and tube growth in Arabidopsis [93]. Pre-rRNA processing protein esf1-like protein is involved in embryo sac egg cell differentiation and karyogamy [94].

## Conclusions

Our study, which has demonstrated that iTRAQ is a powerful technique for performing quantitative phosphoproteomic analyses, represents the first comprehensive phosphoproteomic analysis of cotton fiber differentiation and initiation using a WT and its *fl* mutant. A total of 619 phosphoproteins, including 76 new phosphoproteins, were identified. The 69 differentially phosphorylated proteins were found to be involved in signal transduction, protein modification, carbohydrate metabolic processes, and cell cycle and cell proliferation. Our analysis of WT and *fl* cotton ovule phosphoproteomes sheds light on the post-translational modification role of protein phosphorylation, a major regulator of various biological processes during fiber differentiation and initiation.

### Availability of supporting data

All the raw mass spectra files in LC-MS/MS have been deposited into the publicly accessible database PeptideAtlas and now are available with dataset Identifier PASS00508 (http://www.peptideatlas.org/PASS/PASS00508).

## Electronic supplementary material

Additional file 1: Figure S1: Cotton quantitative phosphoproteomic analysis workflow. WT -3 DPA and WT 0 DPA: Ovules from -3 and 0 DPA developmental stages of Xuzhou 142 WT. *fl -*3 DPA and *fl* 0 DPA: Ovules from -3 and 0 DPA developmental stages of Xuzhou 142 *fl* mutant. A: Peptide sequence identification from peptide backbone fargment ions. B: Quantification from iTRAQ reporter ions. Scale bars: WT -3 DPA and *fl* -3 DPA, 200 μm; WT 0 DPA and *fl* 0 DPA, 20 μm. (TIFF 757 KB)

Additional file 2: **The file contains all original MS/MS spectra of the 830 phosphopeptides identified in this research.** (ZIP 15 MB)

Additional file 3: Table S1: Two sheets were included. Sheet 1: Detected phosphorylated sites. Sheet 2: Phosphoprotein annotations. (XLSX 367 KB)

Additional file 4: Table S2: Comparison of phosphosites conserved between cotton and species in the P^3^DB database. (XLSX 233 KB)

Additional file 5: Table S3: Phosphoprotein Pfam domain information (sheet 1) and phosphosites location in characterized protein domains (sheet 2). (XLSX 232 KB)

Additional file 6: Table S4: Three sheets were included. Distribution of identified phosphoproteins in cellular component (sheet 1), biological process (sheet 2), and molecular function (sheet 3) categories. (XLSX 46 KB)

Additional file 7: Table S5: Novel phosphoproteins (sheet 1) and phosphoproteins with homologs in other species in P^3^DB (sheet 2). (XLSX 9 MB)

Additional file 8: Table S6: Number of transcription factors among the identified phosphoproteins. (XLSX 17 KB)

Additional file 9: Table S7: Sequence alignment of phosphorylation sites and extraction of significantly enriched phosphorylation motifs. (XLSX 61 KB)

Additional file 10: Table S8: Six sheets were included. Motifs identified via Motif-X surrounding localized pSer in nine plant species (sheet 1); pThr residues in nine species (sheet 2); pSer in cotton (sheet 3); pThr residues in cotton (sheet 4); prealigned “phosphor-13-mers” in cotton and nine other species (sheet 5); random protein sequences derived from the genomes of cotton and the nine other species (sheet 6). (XLSX 12 MB)

Additional file 11: Table S9: Five sheets were included. Sheet 1: quantitative analysis of 830 phosphopeptides. Sheet 2: Differentially phosphorylated proteins between WT and *fl* -3 DPA ovules. Sheet 3: Differentially phosphorylated proteins between WT and *fl* 0 DPA ovules. Sheet 4: Differentially phosphorylated proteins between WT 0 DPA and *fl* -3 DPA ovules. Sheet 5: Differentially phosphorylated proteins between *fl* 0 DPA and *fl* -3 DPA ovules. (XLSX 257 KB)

Additional file 12: Figure S2: Molecular functional classification of identified differentially phosphorylated proteins (-3 DPA WT vs. -3 DPA *fl*, and 0 DPA WT vs. 0 DPA *fl*). (TIFF 190 KB)

Additional file 13: Table S10: Molecular functional classification of the 69 differentially phosphorylated proteins identified in this study. (XLSX 13 KB)

Additional file 14: Table S11: The significantly enriched pathways were identified by KOBAS. (XLSX 14 KB)

## Abbreviations

*fl*: Fuzzless-lintless
WT: Wild type
DPA: Days post-anthesis
iTRAQ: Isobaric tags for relative and absolute quantitation
MS/MS: Tandem mass spectrometry
P^3^DB: The plant protein phosphorylation dataBase
LC-MS/MS: Liquid chromatography-tandem mass spectrometry
TiO_2_: Titanium dioxide
SEM: Scanning electron microscopy
DTT: Dithiothreitol
ACN: Acetonitrile
TFA: Trifluoroacetic acid
m/z: Mass-to-charge ratio
CGI: Cotton gene index
GO: Gene ontology
KEGG: Kyoto encyclopedia of genes and genomes
KOBAS: KEGG orthology-based annotation system
RPLP0: 60s acidic ribosomal protein P0
PEPCK: Phosphoenolpyruvate carboxykinase
RhoGAP: Rho GTPase activation protein
BP: Biological process
CC: Cellular component
MF: Molecular function
NR: Non-redundant
TGFB: Transforming growth factor-β receptor kinase
ERKs: Extracellular-signal-regulated kinases
MAPs: Mitogen-activated protein kinases
ROS: Reactive oxygen species
RhoGDIs: Rho-GDP dissociation inhibitors
RNAP II: RNA polymerase II
PPIase: Peptidyl-prolyl cis-trans isomerase
CLD: Cyclophilin-like domain
DCP5: Decapping 5
SUS: Sucrose synthase
FBPase: Fructose-1,6-bisphosphatase
ROPs: Rho proteins of plants.
