# Supplementary figures and images for: Quantitative phosphoproteomic profiling of fiber differentiation and initiation in a fiberless mutant of cotton
Source: BMC Genomics. 2014 Jun 12;15(1):466. doi: 10.1186/1471-2164-15-466 (PMC4070576; doi:10.1186/1471-2164-15-466)

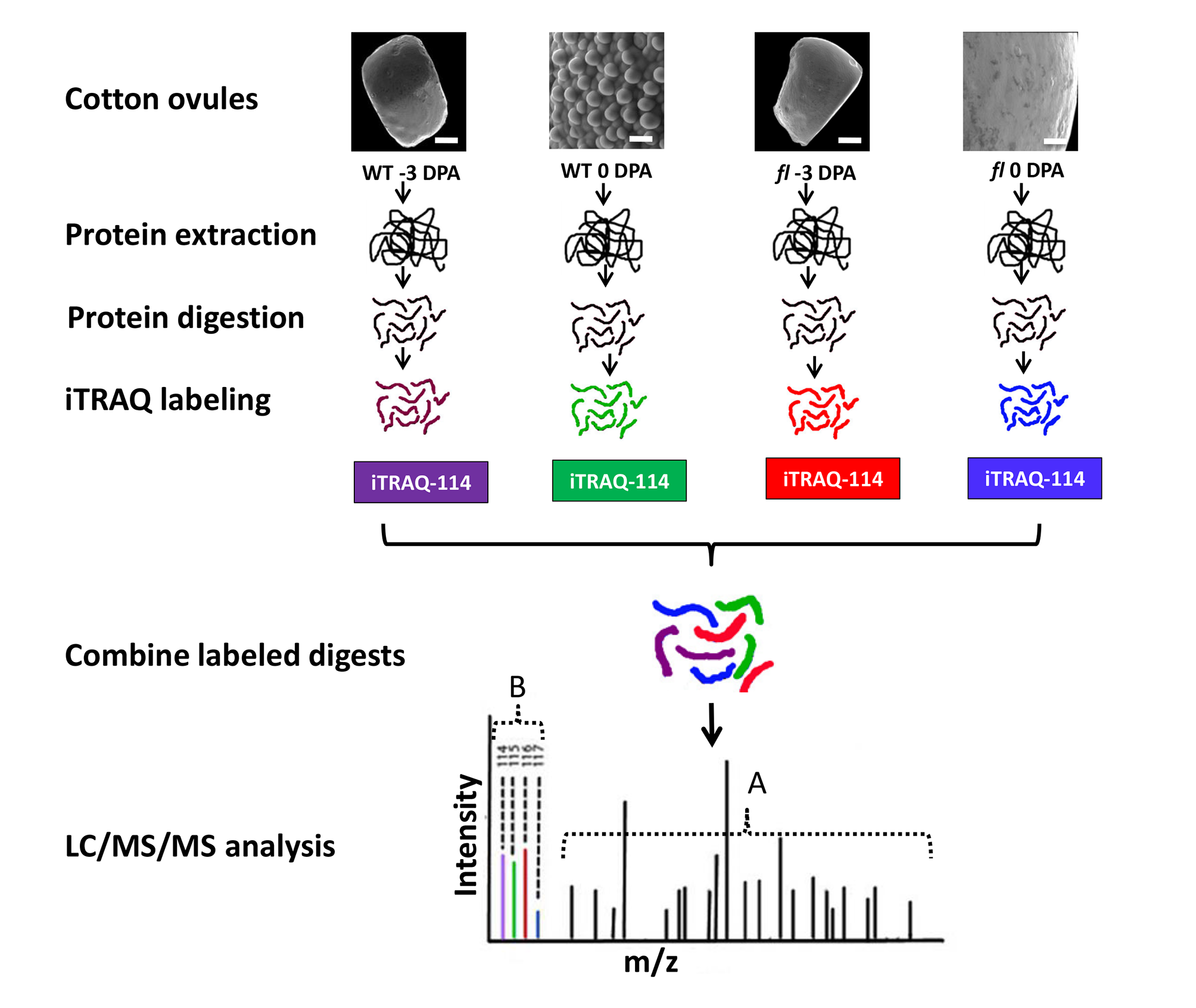

Supplement: Supplementary file 1 — Additional file 1: Figure S1: Cotton quantitative phosphoproteomic analysis workflow. WT -3 DPA and WT 0 DPA: Ovules from -3 and 0 DPA developmental stages of Xuzhou 142 WT. fl -3 DPA and fl 0 DPA: Ovules from -3 and 0 DPA developmental stages of Xuzhou 142 fl mutant. A: Peptide sequence identification from peptide backbone fargment ions. B: Quantification from iTRAQ reporter ions. Scale bars: WT -3 DPA and fl -3 DPA, 200 μm; WT 0 DPA and fl 0 DPA, 20 μm. (TIFF 757 KB) [file 12864_2014_6151_MOESM1_ESM.tiff]

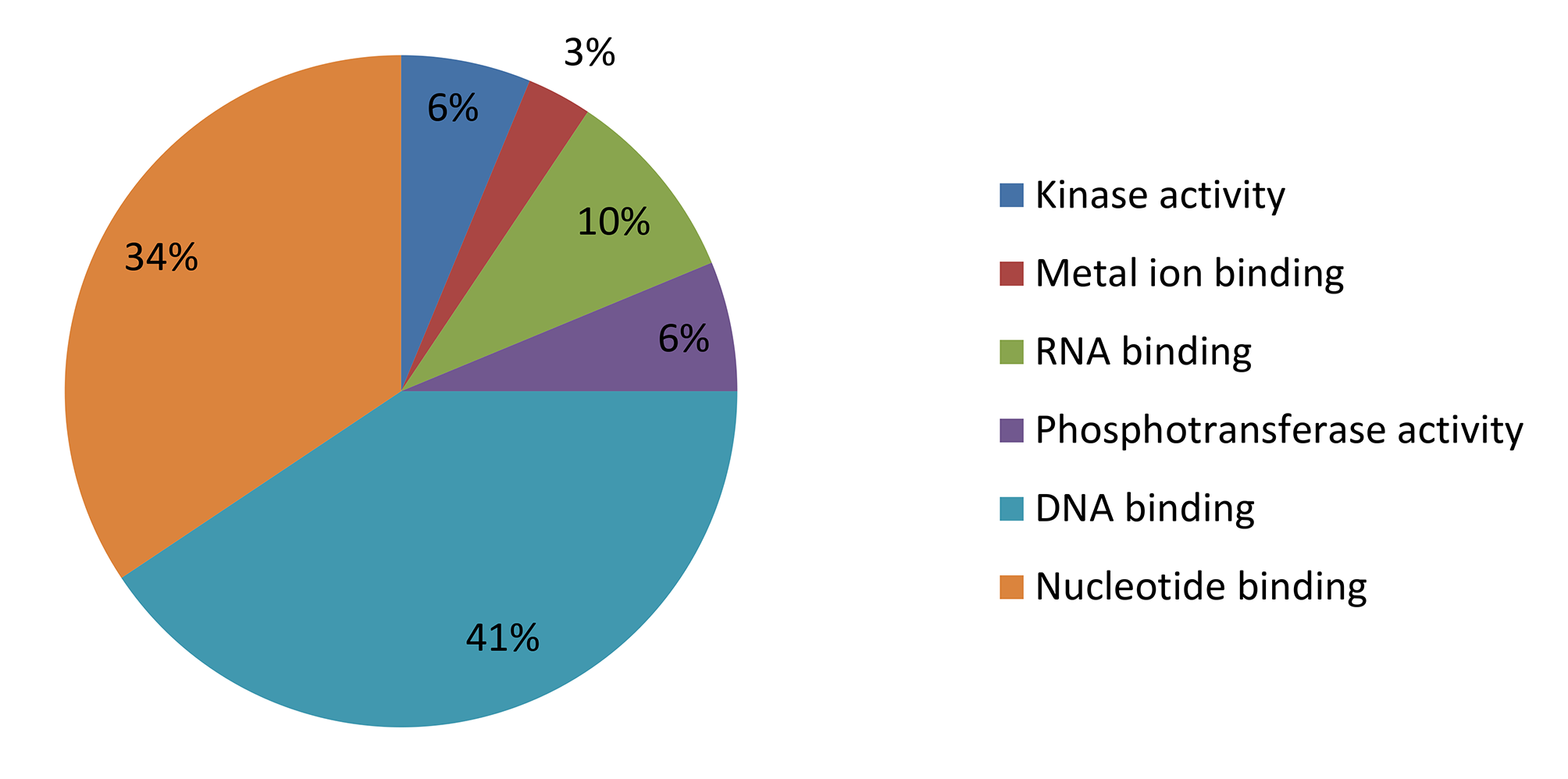

Supplement: Supplementary file 12 — Additional file 12: Figure S2: Molecular functional classification of identified differentially phosphorylated proteins (-3 DPA WT vs. -3 DPA fl, and 0 DPA WT vs. 0 DPA fl). (TIFF 190 KB) [file 12864_2014_6151_MOESM12_ESM.tiff]
